# Dataset on the in vivo absorption characteristics and pigment composition of various phytoplankton species

**DOI:** 10.1016/j.dib.2019.104020

**Published:** 2019-06-02

**Authors:** Lesley A. Clementson, Bozena Wojtasiewicz

**Affiliations:** CSIRO Oceans & Atmosphere, Hobart TAS 7001, Australia

**Keywords:** Phytoplankton, Particulate absorption, Pigments

## Abstract

This paper contains data on the absorption spectra and pigment composition and concentration of 22 phytoplankton species. All phytoplankton cultures were taken from the Australian National Algae Culture Collection. Each absorption spectrum is accompanied by the pigment composition quantified using High Performance Liquid Chromatography (HPLC).

**Table:** Specifications table

Subject area	Biology
More specific subject area	Phytoplankton biology; Marine biology
Type of data	Tables, figures, separate.xlsx (Excel) files with entire measured spectra (raw data) and pigment concentrations
How data was acquired	UV-VIS double-beam spectrophotometer (GBC Scientific Equipment Ltd., Cintra 404); software: Cintral ver. 2.2, HPLC (Waters)
Data format	Analysed, raw
Experimental factors	Phytoplankton cultures (22 species/strains) were taken from the Australian National Algae Culture Collection. The cultures were kept at the exponential growth phase under controlled temperature and light conditions and diluted with fresh medium just before measurements were made.
Experimental features	Absorption measurements were taken with the use of a dual beam spectrophotometer equipped with an integration sphere using the filter-pad method. The absorption coefficients were calculated using the β-correction method described by Mitchell (1990). Pigment composition and concentrations were determined using the HPLC method.
Data source location	Hobart, TAS, Australia
Data accessibility	All data are provided in this article

**Table:** 

**Value of the data** • The dataset contains the characterization of the in vivo absorption properties and pigment composition of phytoplankton monocultures accompanied with full information on the growth temperature and irradiance and cell sizes. • The dataset can be used in the studies of the responses (e.g. changes in the pigment composition, pigment packaging etc.) of phytoplankton species to different growth irradiance. • The dataset can be used in developing models of phytoplankton absorption, primary production and biogeochemical cycling. • The dataset can be a base for theoretical, laboratory or in situ experiments in phytoplankton physiology or ecology and marine bio-optics. • The information on the absorption properties of phytoplankton can be useful in the development of algorithms for remote sensing of specific phytoplankton groups.

## 1 Data

**Fig. 1:**
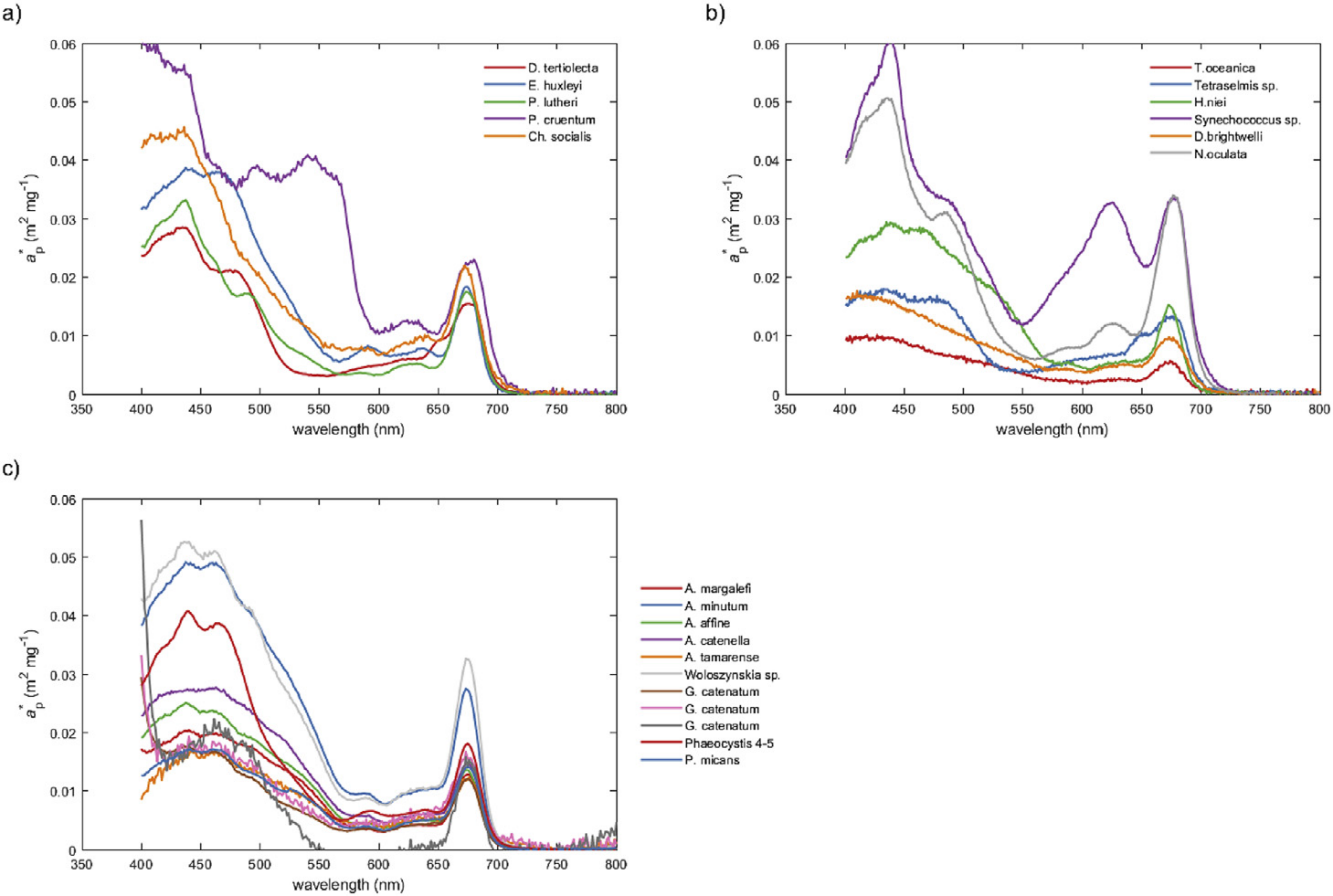
Chlorophyll-specific particulate absorption spectra of phytoplankton monocultures studied in experiment 1 (a), experiment 2 (b), and experiment 3 (c).

**Fig. 2:**
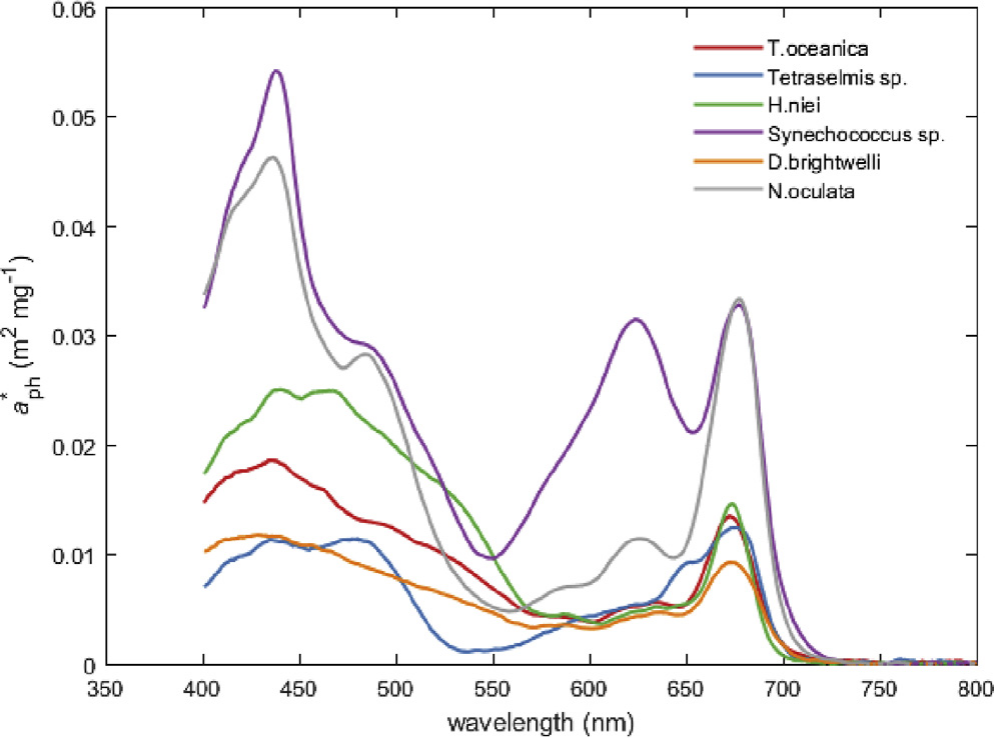
Chlorophyll-specific phytoplankton absorption spectra of phytoplankton monocultures studied in experiment 2.

**Fig. 3:**
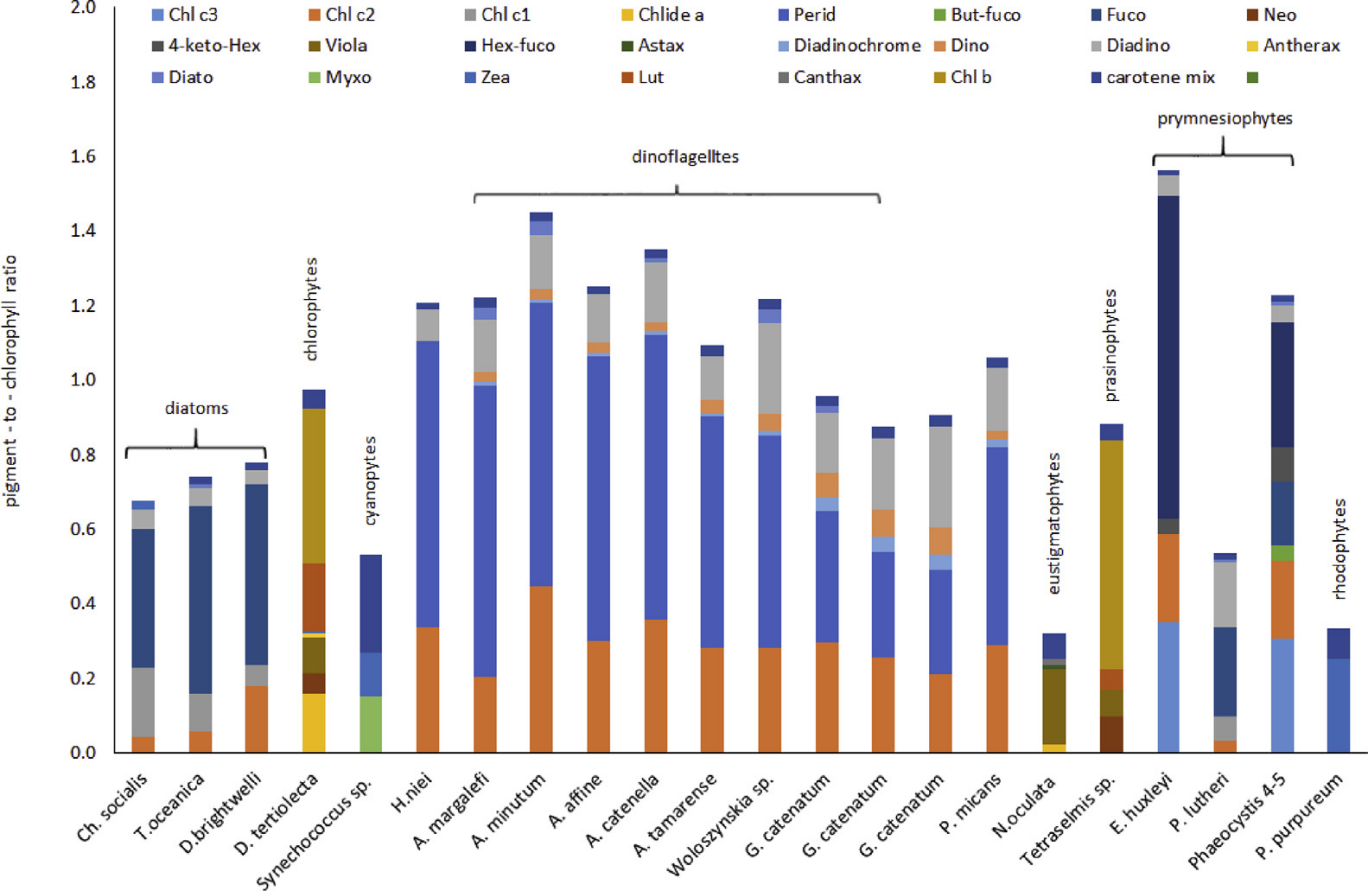
Pigment composition of phytoplankton monocultures.

Data from 3 experiments aiming to determine in vivo absorption coefficients and their pigment composition of chosen phytoplankton species/strains belonging to dinoflagellates (11 strains/species), prymnesiophytes (3), diatoms (3), chlorophytes (1), rhodophytes (1), cyanophytes (1), prasinophytes (1), and eustigmatophytes (1) are presented. The phytoplankton species used in all experiments together with the sizes for most of the strains as well as the growth conditions are given in Table 1. Fig. 1, Fig. 2 present chlorophyll specific total and phytoplankton absorption spectra, respectively. Pigment composition for each of the phytoplankton strains are presented in Fig. 3. Data files contain pigment composition and concentrations (phytoplankton_pigments.xlsx), unsmoothed particulate absorption spectra calculated using the β-correction method described by Mitchell [1] without null-point correction (particulate_absorption_spectra.xlsx) and unsmoothed phytoplankton absorption spectra for cultures used in experiment 2 calculated using the β-correction method described by Mitchell (1990) without null-point correction but smoothed using the running mean filter (phytoplankton_absorption_spectra.xlsx).

**Table 1:** List of analysed phytoplankton species and their cell size and growth irradiance; the size is given as a single diameter (minimum – maximum) for cells of spherical shape and as length (minimum – maximum) by width (minimum – maximum) for cells with elongated shape;^∗^ generic for species not particular strain.

Species	Class	Collection code	Cell size (μm)	Growth irradiance (μmol m^−2^ s^−1^)
**Experiment 1**				
*Dunaliella tertiolecta*	Chlorophyceae	CS-175	10–12	60–80
*Emiliania huxleyi*	Prymnesiophyceae	CS-57	4–8	60–80
*Pavlova lutheri*	Prymnesiophyceae	CS-23	–	60–80
*Porphyridium purpureum*	Rhodophyceae	CS-25	5–8	60–80
*Chaetoceros socialis*	Bacillariophyceae	CS-236	(2–12) x (2–15)^∗^	60–80
**Experiment 2**				
*Thalassiosira oceanica*	Bacillariophyceae	CS-67	8.8–19	50–100
*Tetraselmis* sp.	Prasinophyceae	CS-87	13 × 7	50–100
*Heterocapsa niei*	Dinophyceae	CS-89	20 × 15	50–100
*Synechococcus* sp.	Cyanophyceae	CS-94	1.2–1.5	50–100
*Ditylum brightwelli*	Bacillariophyceae	CS-131	(50–70) x (15 × 20)	50–100
*Nannochloropsis oculata*	Eustigmataceae	CS-189	2–5	50–100
**Experiment 3**				
*Alexandrium margalefi*	Dinophyceae	CS-322	–	80–100
*Alexandrium minutum*	Dinophyceae	CS-323/2	16–25	80–100
*Alexandrium affine*	Dinophyceae	CS-312	–	80–100
*Alexandrium catenella*	Dinophyceae	CS-313/1	26–38	80–100
*Alexandrium tamarense*	Dinophyceae	CS-298	26–38^∗^	80–100
*Woloszynskia* sp.	Dinophyceae	CS-341	23 × 22	80–100
*Gymnodinium catenatum*	Dinophyceae	CS-305	(23–41) x (27–36)	80–100
*Gymnodinium catenatum*	Dinophyceae	CS-309/2	(23–41) x (27–36)	80–100
*Gymnodinium catenatum*	Dinophyceae	CS-309/3	(23–41) x (27–36)	80–100
*Phaeocystis 4-5*	Prymnesiaphyceae	CS - 243	–	6
*Prorocentrum micans*	Dinophyceae	CS-27	–	80–100

## 2 Experimental design, materials, and methods

All phytoplankton cultures (22 species) were taken from the Australian National Algae Culture Collection. The cultures were kept at the exponential growth phase and diluted with fresh medium just before measurements. All cultures were grown under 12/12 h light/dark cycle. The growth light conditions are given in Table 1. The cultures were grown at 20 °C, apart from *Phaeocystis* sp. CS-243 strain, which was grown at 5 °C.

Absorption was measured using a dual beam spectrophotometer (GBC Scientific Equipment Ltd., Cintra 404) equipped with an integration sphere using the filter-pad method with the sample filter placed at the entrance of the integrating sphere and a moistened filter placed in the reference beam. The baseline was determined for both filters saturated in the growth medium. The spectra were measured over the 400–800 nm spectral range in 1.3 nm (experiments 1 & 3) and 0.85 nm (experiment 2) increments. The absorbance obtained from the measurements was converted into the total particulate absorption coefficient (*a*_p_(λ), m^−1^) by multiplying the appropriate baseline-corrected optical density values of the sample by 2.3 and dividing by the optical path length/cuvette thickness (0.01 m). The absorption coefficients were calculated using the β-correction method described by [1]. In the case of the second experiment after the first measurement the pigments on the filter were extracted using methanol [2] and the absorbance of the filter with remaining particles was measured again to derive the nonalgal (NAP) absorption coefficient (*a*_NAP_(λ), m^−1^) which was calculated in the same way as *a*_p_(λ). The phytoplankton absorption coefficient (*a*_ph_(λ), m^−1^) was calculated as the difference between *a*_p_(λ) and *a*_NAP_(λ).

The *a*_p_(λ) results from first and third experiment can be converted into *a*_ph_(λ) assuming exponential shape of *a*_NAP_(λ) using a method described by [3].

Data presented in Fig. 1, Fig. 2 were null-point corrected by subtracting the absorption coefficient value at 750 nm assuming no absorption in the NIR region of the spectrum [e.g., [4]].

Pigment composition and concentrations were determined using the HPLC CSIRO method described in [5] using a C_8_ column and binary gradient system with an elevated column temperature (Waters). Pigments were identified by their retention time and their absorption spectra from the photo-diode array detector. The pigment concentrations were determined through peak integration (Empower^©^ software).
